# Photobiomodulation modulates peripheral and central expression of inflammation-related genes following carrageenan-induced inflammation in rats

**DOI:** 10.1007/s10103-026-05096-9

**Published:** 2026-11-10

**Authors:** Carlos Alberto-Silva, Giulia Nicolle Jácome Cartaxo, Regiane dos Santos Feliciano, Bruna Calixto de Jesus, José Antônio Silva-Junior, Maricilia Silva Costa

**Affiliations:** 1 Natural and Humanities Sciences Center, Experimental Morphophysiology Laboratory, Universidade Federal do ABC, Santo André, Brazil; 2 Instituto de Pesquisa e Desenvolvimento – IP&D, Universidade do Vale do Paraíba, São José dos Campos, Brazil; 3 Postgraduate Program in Biophotonics applied to Health Sciences, Universidade Nove de Julho, São Paulo, Brazil

**Keywords:** Photobiomodulation, Peripheral inflammation, Kinin receptors, Interleukin-6, Interleukin-10, Prostacyclin receptor

## Abstract

Peripheral inflammation can induce molecular changes in central nervous system through immune–brain communication. This study investigated whether photobiomodulation (PBM) modulates the temporal mRNA expression of pro- and anti-inflammatory cytokines and receptors involved in prostacyclin and kinin signaling in peripheral and central tissues following carrageenan-induced inflammation in rats. Male Wistar rats received subplantar carrageenan injections (1 mg/paw) and were treated or not with PBM (660 nm) 1 h after carrageenan administration. Animals were euthanized at 1, 3, or 6 h, and mRNA expression of IL-6, IL-10, prostacyclin receptor (IP), and kinin B1 and B2 receptors was quantified by qRT-PCR in subplantar and brain tissues. IL-6 and IP receptor mRNA expression showed their highest levels at 6 h among the carrageenan-treated groups and were reduced by PBM at this time point in both subplantar and brain tissues. In the paw, B1 receptor expression showed its highest level at 3 h among the time points evaluated and was reduced by PBM, whereas B2 receptor expression showed its greatest increase at 6 h. In the brain, PBM reduced B1 and B2 receptor expression at 6 h. IL-10 expression increased in the paw, remaining elevated in PBM-treated animals. In the brain, IL-10 expression was higher at 3 h than at 6 h following carrageenan administration, whereas PBM maintained elevated IL-10 expression at 6 h. Carrageenan-induced peripheral inflammation was accompanied by time-dependent changes in inflammation- and nociception-related gene expression in brain tissue. Localized PBM modulated these peripheral and central transcriptional responses by reducing IL-6, IP, and kinin receptor expression at specific time points while maintaining elevated IL-10 expression. These findings extend previous observations by showing modulation of cytokine, prostacyclin, and kinin signaling-related gene expression following localized PBM.

## Introduction

The bidirectional communication between the peripheral immune system and the central nervous system (CNS) is increasingly recognized as an important determinant of neurological function and health. Accumulating evidence indicates that inflammation initiated in peripheral tissues can influence brain function and trigger central inflammatory responses, even when the initial insult is localized. Such peripheral–central interactions have been associated with alterations in higher-order cognitive functions and other neurological outcomes [1]. Although acute conditions such as systemic infections or traumatic injuries generally elicit transient inflammatory responses [2], peripheral immune activation can also influence CNS function and contribute to neuroinflammatory processes. Neuroinflammation represents an important component of peripheral–brain communication and may contribute to neurological dysfunction following peripheral inflammatory insults [3]. Peripheral inflammatory challenges have also been shown to induce changes in glial activity, including the activation of microglia and astrocytes, which transition into reactive states following peripheral immune stimulation [4]. A well-established experimental approach for investigating peripheral inflammatory responses and their consequences for central signaling is the carrageenan-induced paw inflammation model, which reliably produces acute inflammation and hyperalgesia and has been extensively used to investigate the molecular mechanisms underlying inflammatory responses and pain [5].

Subplantar injection of carrageenan induces a well-characterized biphasic inflammatory response involving the sequential release and action of several inflammatory mediators. The early phase is associated predominantly with the release of vasoactive mediators, including histamine and bradykinin, whereas the later phase involves increased production of prostanoids and pro-inflammatory cytokines, including tumor necrosis factor-alpha (TNF-α), interleukin-1 beta (IL-1β), and interleukin-6 (IL-6) in the inflamed tissue [6–8]. The kinin and prostanoid systems play important roles in inflammatory pain, with bradykinin signaling involving B2 receptors and, particularly under inflammatory conditions, inducible B1 receptors [9]. Prostacyclin signaling through the IP receptor also contributes to inflammatory nociception and pain sensitization [10]. Importantly, the inflammatory consequences of carrageenan-induced peripheral injury are not restricted to the site of injection. Experimental studies have demonstrated that this localized inflammatory challenge can induce changes in inflammatory mediators and related signaling pathways within the CNS, indicating that peripheral inflammation can engage mechanisms of peripheral–brain communication [11–13].

In this context, photobiomodulation (PBM) has emerged as a promising non-invasive approach for modulating inflammatory responses. Increasing evidence indicates that PBM can influence multiple cellular and molecular pathways involved in inflammation, including the regulation of cytokine production and signaling associated with inflammatory and pain responses [14–16]. Importantly, our previous study demonstrated that 660 nm PBM reduces IL-1β, mPGES-1, and mPGES-2 mRNA expression in both inflamed paw and brain tissues, whereas EP receptor expression was not significantly affected [17]. Earlier findings from this experimental model also demonstrated reduced COX-2 mRNA expression following PBM [18]. These findings provided evidence that localized PBM can modulate inflammatory signaling beyond the site of irradiation, including within the CNS. Building on these findings, the present study investigated whether localized PBM can modulate additional components of the peripheral and central inflammatory response following carrageenan-induced peripheral inflammation. Specifically, we evaluated the temporal expression of IL-6 and the anti-inflammatory cytokine IL-10, together with the prostacyclin (IP) receptor and kinin B1 and B2 receptors, in both paw and brain tissues. Together, these targets provide a complementary view of the inflammatory response by surrounding pro- and anti-inflammatory cytokine signaling combined with receptor systems involved in kinin- and prostanoid-mediated inflammatory processes. By comparing their expression profiles across peripheral and central tissues and different stages of inflammation, we investigated whether PBM produces molecular effects in both peripheral and central tissues beyond the previously characterized regulation of IL-1β, COX-2, mPGES-1, and mPGES-2 [17]. We hypothesized that localized 660 nm PBM would modulate inflammation-associated gene expression by reducing selected pro-inflammatory and nociception-related mediators while preserving the expression of the regulatory cytokine IL-10 in peripheral and brain tissues.

Previous studies from our group demonstrated that PBM can modulate the expression of selected inflammatory mediators in peripheral and central tissues following carrageenan-induced inflammation. However, the temporal relationship between peripheral and central transcriptional responses and the broader involvement of inflammatory, regulatory, and nociception-related targets remain incompletely characterized. The present study extends these previous findings by incorporating IL-6, IL-10, and the bradykinin B1 and B2 receptors into a temporal analysis of peripheral and whole-brain transcriptional responses, while evaluating PBM-associated modulation at defined post-carrageenan time points. This approach provides a broader view of the molecular response to localized PBM across peripheral and central compartments.

## Materials and methods

### Chemicals and animals

Male Wistar rats aged 5–7 weeks and weighing 150–200 g were obtained from the Central Animal House of the -----------. All experimental procedures were approved by the Ethics Committee on Animal Use of ------------------- (protocol ------------). Animals were housed six per cage at 25 °C and 50% ± 15% relative humidity under a 12-h light/dark cycle with constant ventilation and had ad libitum access to standardized laboratory chow and water.

### Peripheral inflammation model and experimental design

Peripheral inflammation was induced by subplantar administration of carrageenan, as previously described [17]. Thirty-six male Wistar rats were randomly allocated to six experimental groups (*n* = 6 per group). Under anesthesia with 1% halothane (Sigma Chemical Co., St. Louis, MO, USA), animals received a subplantar injection of approximately 100 µL of carrageenan solution (1 mg/paw; Sigma Chemical Co., St. Louis, MO, USA) diluted in 0.90% NaCl into the left hind paw using a 27-gauge needle.

The experimental groups were defined according to carrageenan administration, PBM treatment, and time of euthanasia. The Control group received 0.90% NaCl and was euthanized 6 h after injection. The Carr-1, Carr-3, and Carr-6 groups received carrageenan and were euthanized 1, 3, and 6 h after injection, respectively. In the PBM-treated groups, PBM was administered locally to the carrageenan-injected paw 1 h after carrageenan administration. These animals were euthanized either 3 h (Carr + PBM-3) or 6 h (Carr + PBM-6) after carrageenan injection. Thus, the numerical designation of each experimental group refers to the time of euthanasia and tissue collection after carrageenan administration, rather than to the time of PBM application.

The experimental design was intended to characterize the temporal pattern of transcriptional responses following carrageenan-induced peripheral inflammation and to evaluate PBM-associated modulation at selected post-carrageenan time points. Accordingly, PBM-treated and untreated carrageenan groups were compared at matched tissue-collection times (Carr-3 versus Carr + PBM-3 and Carr-6 versus Carr + PBM-6).

**Fig. 1:**
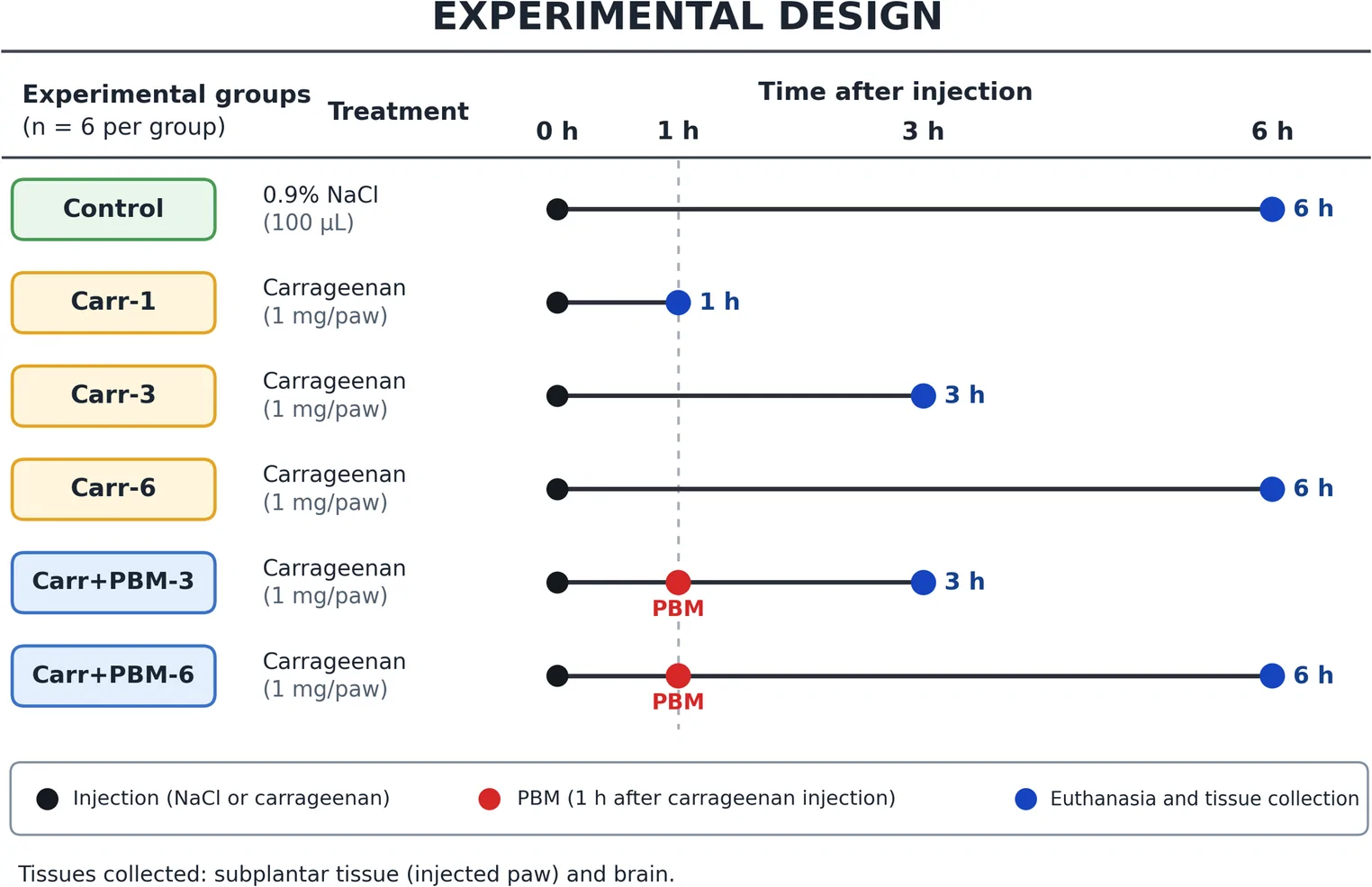
Experimental design and timeline. Male Wistar rats were allocated into six groups (*n* = 6/group) according to carrageenan administration, PBM treatment, and euthanasia time (1, 3, or 6 h). The control group received a subplantar injection of saline and was euthanized at 6 h. Carrageenan-treated animals received 1 mg/paw and were euthanized at 1, 3, or 6 h. PBM (660 nm) was applied to the inflamed paw 1 h after carrageenan injection, and PBM-treated animals were euthanized at 3–6 h. Subplantar and total brain tissues were collected immediately after euthanasia. PBM, photobiomodulation; Carr, carrageenan

The overall experimental design and timeline are summarized in Fig. 1. Animals were euthanized by CO₂ asphyxiation. Subplantar tissue from the injected paw and brain tissue were rapidly collected on a cold plate, immediately frozen in liquid nitrogen, and stored at − 80 °C until RNA extraction.

### Photobiomodulation parameters

Photobiomodulation was performed using a diode laser emitting at 660 nm with a nominal output power of 30 mW (Laser Unit, Kondortech, São Carlos, SP, Brazil), operated in continuous-wave mode. The laser beam covered an area of 0.785 cm², corresponding to a nominal power density of 38.2 mW/cm², and was applied to the plantar surface of the inflamed paw in non-contact mode, at a distance of 1.2 cm, with no intervening medium between the laser source and the paw. The irradiation parameters corresponded to an energy density of 7.5 J/cm², a nominal total energy of 5.88 J, and a total irradiation time of 196 s per application, as previously described [17]. The output power corresponded to the nominal value specified for the device and was not independently measured at the treatment surface.

### Quantitative real-time PCR (qRT-PCR)

Total RNA was extracted from subplantar and brain tissues using TRIzol^®^ Reagent (Life Technologies, Carlsbad, CA, USA) according to the manufacturer’s instructions. RNA concentration and purity were determined with a NanoDrop ND-2000 spectrophotometer (NanoDrop Products, Wilmington, DE, USA), and only samples with A260/A280 between 1.8 and 2.0 and A260/A230 of ~ 1.8 were used. RNA integrity was assessed by 1% agarose gel electrophoresis. To remove genomic DNA, 1 µg of total RNA was treated with RNase-free DNase I (Invitrogen, Carlsbad, CA, USA) for 15 min at 37 °C, followed by inactivation at 65 °C for 10 min. Complementary DNA (cDNA) was synthesized using SuperScript^®^ IV Reverse Transcriptase (Invitrogen) with oligo d(T)20 primers (65 °C for 5 min; 55 °C for 10 min; 80 °C for 10 min), followed by RNase H treatment (37 °C for 20 min). qRT-PCR was performed in duplicate using TaqMan™ Universal Fast Master Mix (2×) and TaqMan Gene Expression Assays (Applied Biosystems, Carlsbad, CA, USA) for IL-6, IL-10, IP receptor (Ptgir), B1 receptor (Bdkrb1), B2 receptor (Bdkrb2), and 18 S rRNA. Reactions (10 µL final volume) were run on an ABI 7500 Fast system with an initial step at 95 °C for 20 s, followed by 40 cycles of 95 °C for 3 s and 60 °C for 30 s. Fluorescence was normalized to the ROX passive reference. No-template controls were included in each run. Data was processed with SDS 1.4 (Applied Biosystems). Gene expression was normalized to 18 S rRNA and calculated by the 2^−ΔΔCt method. ΔCt was calculated for each sample as the mean Ct of the target gene minus the mean Ct of 18 S rRNA (ΔCt = Ct_target − Ct_18S), and ΔΔCt as the ΔCt of each sample minus the mean ΔCt of the saline-treated control group. Samples were analysed in duplicate.

### Statistical analysis

Data are presented as mean ± standard deviation (SD), with *n* = 6 animals per experimental group. Statistical significance was assessed using one-way analysis of variance (ANOVA), followed by the Tukey–Kramer post-hoc test for multiple comparisons. Each gene and tissue was analyzed separately. Temporal comparisons were performed among the Carr-1, Carr-3, and Carr-6 groups, whereas PBM-associated differences were evaluated between PBM-treated and untreated carrageenan groups at matched tissue-collection times. Differences were considered statistically significant at *P* < 0.05. Statistical analyses were performed using GraphPad Prism 7.0 (GraphPad Software, La Jolla, CA, USA).

## Results

### PBM attenuates IP receptor and IL-6 mRNA expression in subplantar and brain tissues

**Fig. 2:**
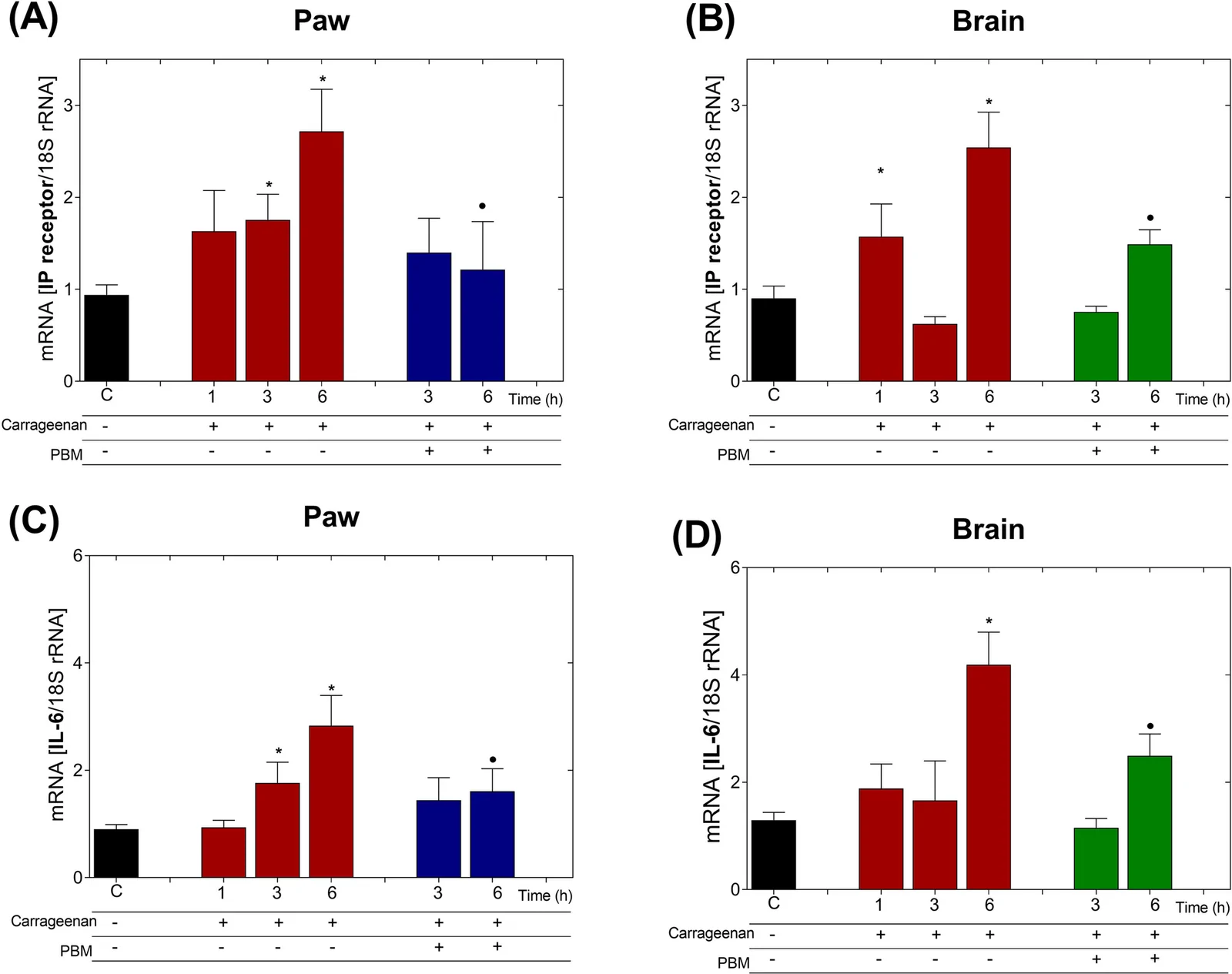
PBM attenuates IP receptor and IL-6 mRNA expression following carrageenan-induced peripheral inflammation. IP receptor mRNA expression was evaluated in subplantar tissue (**A**) and total brain tissue (**B**), whereas IL-6 mRNA expression was evaluated in subplantar tissue (**C**) and total brain tissue (**D**). Gene expression was quantified by qRT-PCR, normalized to 18 S rRNA expression, and presented as relative mRNA expression. Data are presented as mean ± SD of six animals per experimental group (*n* = 6) and were analyzed by one-way ANOVA followed by the Tukey–Kramer post hoc test. Different symbols indicate significant differences (*p* < 0.05): *, compared with the saline-treated control group; ●, compared with the carrageenan group at 6 h. C, saline-treated control; PBM, photobiomodulation

In the paw, IP receptor mRNA expression showed a time-dependent pattern among carrageenan-treated groups, with the highest level observed at 6 h post-injection (Fig. 2a). At this time point, PBM significantly reduced IP receptor expression compared with the corresponding carrageenan group. IP receptor mRNA expression showed a different temporal pattern in the brain, with higher expression at 1 h, lower levels at 3 h, and higher levels again at 6 h. PBM significantly reduced IP receptor expression at 6 h compared with Carr-6 (Fig. 2b). A similar late response was observed for IL-6. In the paw, IL-6 mRNA expression showed higher levels at 3 and 6 h among the carrageenan-treated groups, with the highest expression at 6 h. PBM significantly reduced IL-6 expression at 6 h compared with Carr-6 (Fig. 2c). In the brain, IL-6 expression showed its highest level at 6 h among the carrageenan-treated groups and was significantly reduced by PBM at the same time point (Fig. 2d).

### PBM maintains elevated IL-10 mRNA expression following carrageenan-induced inflammation

**Fig. 3:**
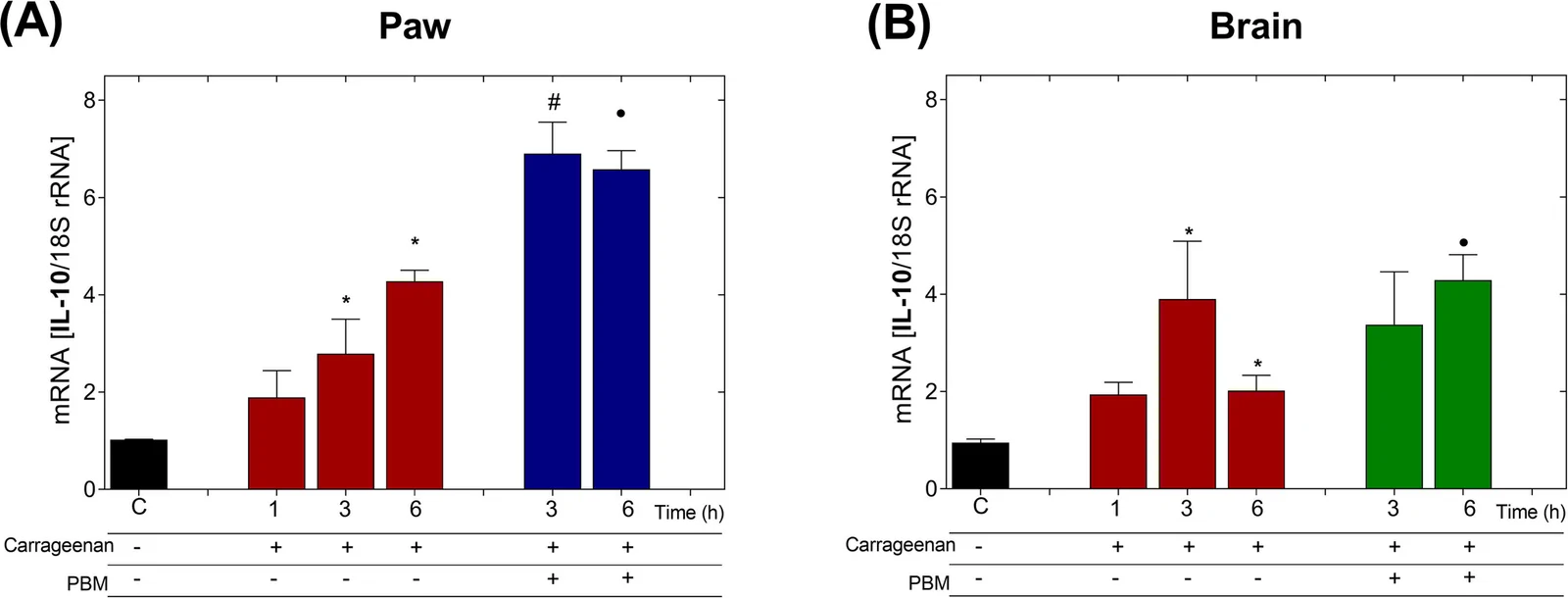
PBM preserves IL-10 mRNA expression during carrageenan-induced peripheral inflammation. IL-10 expression showed higher levels in subplantar tissue at 3 and 6 h among the carrageenan-treated groups and remained elevated in PBM-treated animals (**A**). In total brain tissue, IL-10 expression was higher at 3 h than at 6 h among the carrageenan-treated groups, whereas PBM maintained elevated expression at the later time point (**B**). IL-10 mRNA was quantified by qRT-PCR and normalized to 18 S rRNA. Results are expressed as mean ± SD for six animals per group. Differences among experimental groups were assessed by one-way ANOVA followed by the Tukey–Kramer post hoc test. Different symbols indicate significant differences (*p* < 0.05): *, versus the saline-treated control group; #, versus the carrageenan group at 3 h; and ●, versus the carrageenan group at 6 h. C, saline-treated control; PBM, photobiomodulation

IL-10 mRNA expression in the paw showed higher levels at 3 and 6 h among the carrageenan-treated groups (Fig. 3a). In PBM-treated animals, IL-10 expression remained elevated at both time points. However, a different temporal profile was observed in the brain, with higher IL-10 expression at 3 h and lower expression at 6 h among the carrageenan-treated groups. In animals treated with PBM, IL-10 expression remained elevated at 6 h (Fig. 3b).

### PBM differentially modulates kinin B1 and B2 receptor mRNA expression in subplantar and brain tissues

**Fig. 4:**
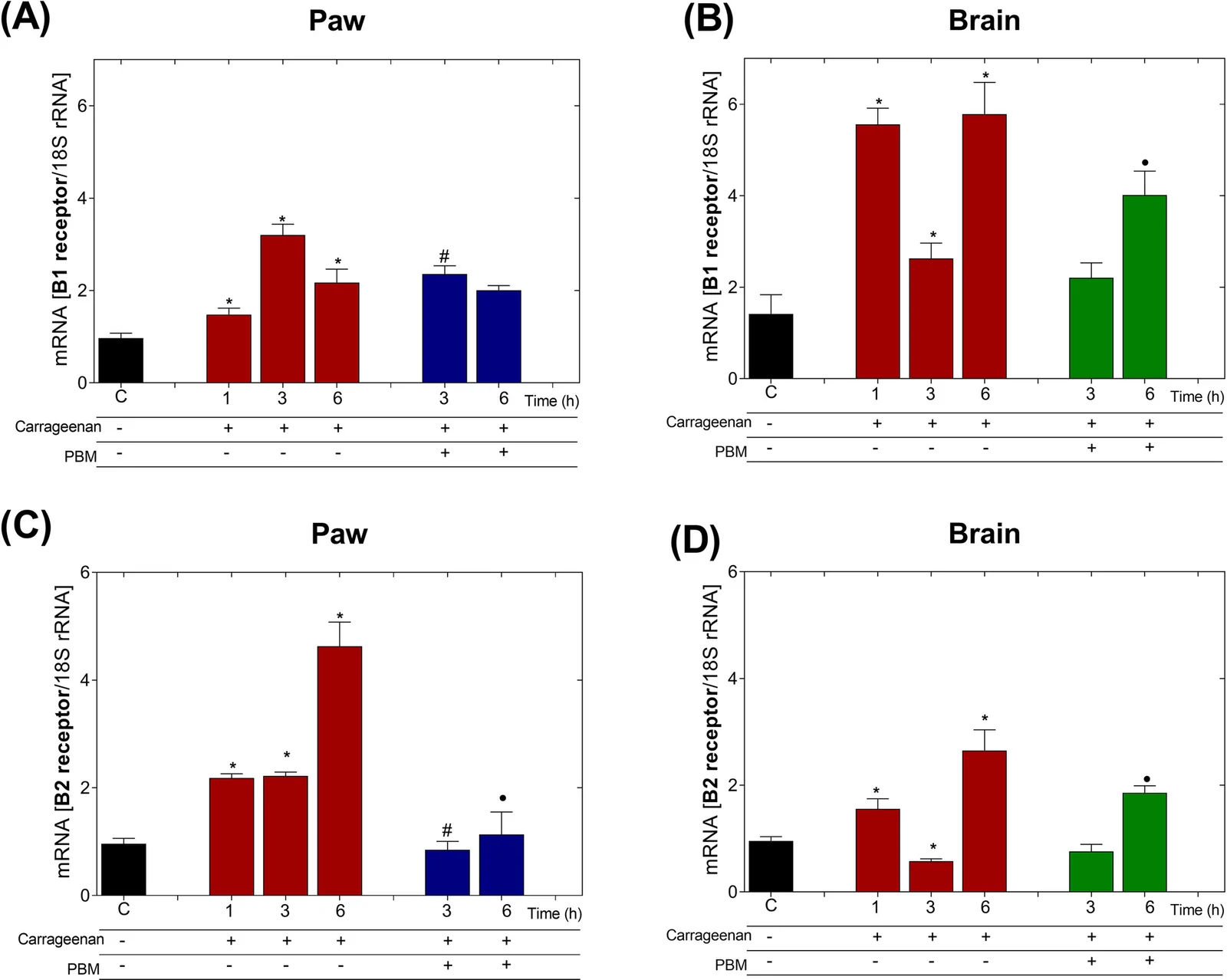
PBM differentially modulates the temporal expression of kinin B1 and B2 receptors in subplantar and total brain tissues. B1 receptor expression is presented for subplantar tissue (**A**) and total brain tissue (**B**), whereas B2 receptor expression is presented for subplantar tissue (**C**) and total brain tissue (**D**). In the paw, B1 receptor expression reached its highest level at 3 h, whereas B2 receptor expression reached its highest level at 6 h among the carrageenan-treated groups. In total brain tissue, both receptors exhibited time-dependent expression profiles and were significantly reduced by PBM at 6 h. Relative mRNA expression was determined by qRT-PCR using 18 S rRNA as the reference gene. Data represent mean ± SD (*n* = 6 animals/group) and were analyzed by one-way ANOVA with the Tukey–Kramer post hoc test. Different symbols indicate significant differences (*p* < 0.05): *, versus the saline-treated control group; #, versus the carrageenan group at 3 h; and ●, versus the carrageenan group at 6 h. C, saline-treated control; PBM, photobiomodulation

Kinin B1 receptor mRNA expression in the paw showed a time-dependent pattern among the carrageenan-treated groups, reaching its highest level at 3 h (Fig. 4a). At this time point, PBM significantly reduced B1 receptor expression compared with Carr-3. In the brain, B1 receptor expression showed a biphasic pattern, with higher expression at 1 h, lower levels at 3 h, and higher levels again at 6 h. PBM significantly reduced B1 receptor expression at 6 h compared with Carr-6 (Fig. 4b). Kinin B2 receptor mRNA expression in the paw showed its highest level at 6 h among the carrageenan-treated groups (Fig. 4c). In PBM-treated animals, B2 receptor expression at 3 and 6 h remained below the marked increase observed in the Carr-6 group. In the brain, B2 receptor expression was higher at 1 h, lower at 3 h, and reached its highest level at 6 h among the carrageenan-treated groups. PBM significantly reduced B2 receptor expression at 6 h compared with Carr-6 (Fig. 4d).

## Discussion

**Fig. 5:**
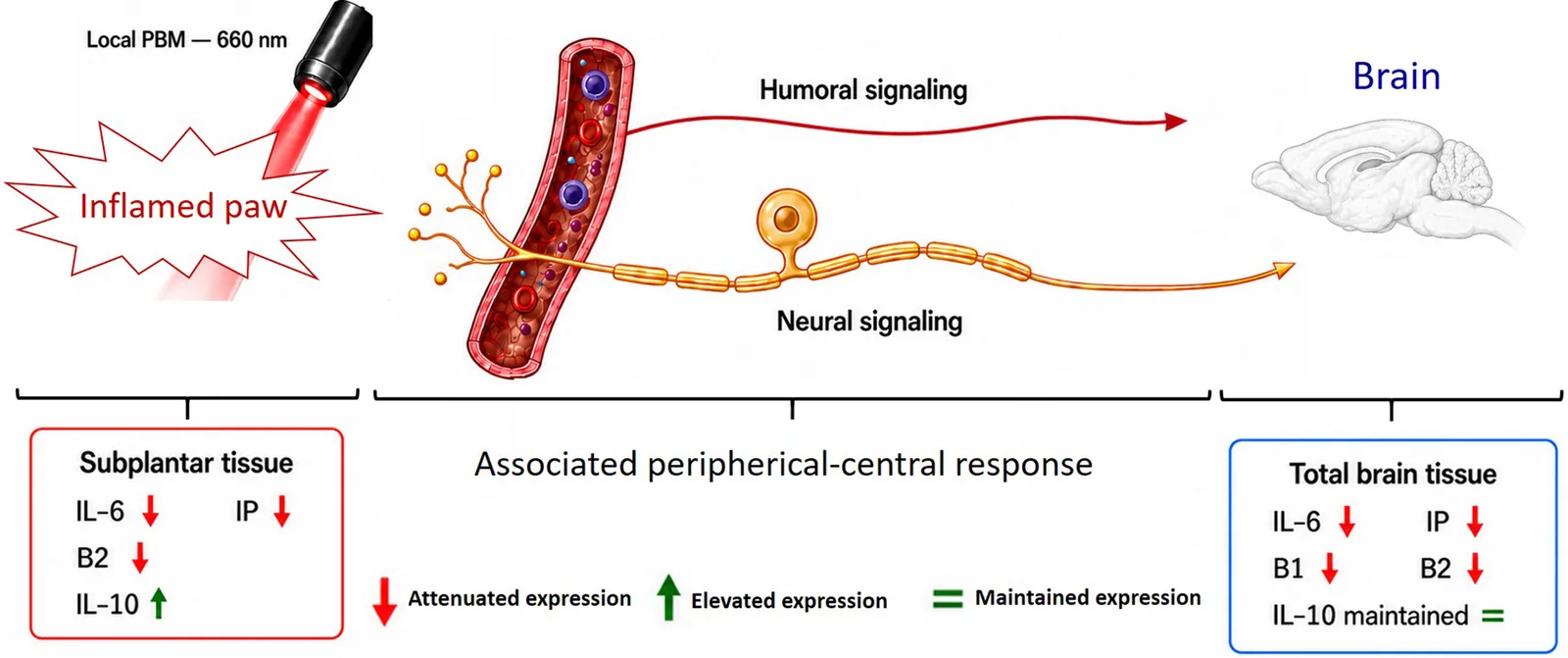
Integrated peripheral and central transcriptional responses associated with localized photobiomodulation following carrageenan-induced inflammation. PBM was applied locally to the inflamed paw 1 h after carrageenan administration. The schematic summarizes the responses observed at the final experimental time point, 6 h after inflammation induction, corresponding to approximately 5 h after PBM. At this time point, PBM attenuated IL-6, IP receptor, and kinin B2 receptor mRNA expression in subplantar tissue. Subplantar IL-10 mRNA expression remained elevated relative to the saline-treated control. In total brain tissue, PBM attenuated IL-6, IP receptor, and kinin B1 and B2 receptor mRNA expression, while IL-10 mRNA expression remained elevated. Humoral and neural signalling are depicted as plausible routes of peripheral–central communication but were not directly investigated in the present study. Downward arrows indicate attenuated mRNA expression, the upward arrow indicates elevated mRNA expression, and the equals sign indicates expression that remained elevated following PBM. PBM, photobiomodulation; IP, prostacyclin receptor; B1 and B2, kinin receptors

The principal finding of this study is that localized PBM produced time-dependent changes in inflammation- and nociception-related gene expression in both the inflamed paw and total brain tissue. Specifically, PBM attenuated IL-6, IP, and kinin B1 and B2 receptor mRNA expression at specific time points while maintaining elevated expression of the regulatory cytokine IL-10. These results indicate that the molecular response associated with localized PBM is not restricted to the irradiated tissue and involves multiple components of the peripheral and central inflammatory response. An integrated overview of the peripheral and central transcriptional responses observed at the final experimental time point is presented in Fig. 5. These findings are consistent with evidence that peripheral inflammatory stimuli can be accompanied by molecular changes in the CNS [1, 3].

Peripheral inflammatory signals can reach the brain through neural and humoral pathways, providing a biological basis for the central responses observed following a peripheral inflammatory challenge [19]. However, these potential routes of peripheral–central communication were not directly investigated in the present study. Previous studies using carrageenan-induced peripheral inflammation have also demonstrated changes in inflammatory mediators within the CNS, further supporting the communication between peripheral inflammatory events and central molecular responses [11, 12, 17]. The present findings extend these previous observations by showing changes in the expression of genes related to pro- and anti-inflammatory **c**ytokines and components of the kinin–kallikrein system in the molecular response associated with localized PBM. These findings are consistent with the immunomodulatory effects of PBM reported in different experimental models [14–16, 20].

The kinin–kallikrein system was markedly modulated by PBM, particularly through changes in B1 and B2 receptor expression. These receptors play important roles in inflammatory and nociceptive responses, and their expression may vary according to the inflammatory condition and its temporal evolution [9]. In the paw, kinin receptor expression showed a time-dependent response to carrageenan, with B1 receptor mRNA reaching its highest level at 3 h, whereas B2 receptor mRNA showed its greatest increase at 6 h. PBM reduced B1 receptor expression at 3 h, while B2 receptor expression at 3 and 6 h remained below the marked increase observed in the Carr-6 group. These findings indicate that PBM modulates kinin receptor expression during the progression of the peripheral inflammatory response. In agreement with these results, de Oliveira et al. [21], analyzing inflammatory and pain mediators after papain-induced osteoarthritis in rats, observed attenuated mRNA and protein levels of kinin B1 and B2 receptors following PBM. Changes in kinin receptor expression were also observed in the brain, where PBM reduced B1 and B2 receptor mRNA expression at 6 h. This finding suggests that the effects associated with localized PBM are accompanied by molecular changes beyond the irradiated tissue, including the modulation of inflammation-associated pathways in the CNS. Manchini et al. [22] also observed reduced B1 receptor expression after PBM in inflamed subplantar muscle, but increased B2 receptor mRNA expression 72 h after myocardial infarction. Such differences may be related to the experimental model, tissue environment, and time after injury. In addition, previous evidence demonstrates functional cross-talk between B1 and B2 receptors, including B2-mediated regulation of B1 receptor signaling and desensitization, indicating that the activity of these receptors may not occur independently under inflammatory conditions [23]. Thus, the different temporal profiles observed for B1 and B2 receptor expression in the present study may reflect the dynamic regulation of the kinin system during the inflammatory response.

The central molecular response observed in this study was also evident for IL-6 and the IP receptor. IL-6 is a pleiotropic cytokine involved in the regulation and progression of inflammatory responses [24] and has also been proposed as an important mediator of inflammatory communication between peripheral tissues and the CNS. In a carrageenan-induced inflammatory model, Oka et al. [12] demonstrated that circulating IL-6 may act as a messenger of inflammatory information from the peripheral inflammatory site to the CNS. In the present study, IL-6 mRNA expression increased markedly at 6 h in both the paw and brain, whereas PBM significantly reduced its expression at this time point. This parallel response further supports the close relationship between peripheral inflammatory events and central molecular changes and indicates that PBM can modulate IL-6 expression at both levels. A similar effect was observed for the IP receptor. Prostacyclin signaling through the IP receptor plays an important role in inflammatory pain and contributes to nociceptor sensitization [10]. In the present study, IP receptor mRNA expression increased at 6 h after carrageenan administration and was significantly reduced by PBM in both paw and brain tissues. Therefore, modulation of IP receptor expression may be associated with the effects of PBM on genes related to inflammatory and nociceptive signaling. These findings are supported by Chagas et al. [25], who demonstrated increased IP receptor mRNA expression in both subplantar and brain tissues 6 h after carrageenan-induced peripheral inflammation and a significant reduction following 660 nm irradiation. Together with the present findings, these results reinforce the involvement of prostanoid signaling in the molecular response to PBM during carrageenan-induced inflammation.

Another relevant finding was the modulation of IL-10, an anti-inflammatory cytokine involved in the control and resolution of inflammatory responses. Previous studies have demonstrated that photobiomodulation can modulate IL-10 production in different inflammatory conditions [26–28]. In the paw, IL-10 mRNA expression showed higher levels at 3 and 6 h among the carrageenan-treated groups and remained elevated in PBM-treated animals at 3 and 6 h. In the brain, IL-10 expression was higher at 3 h than at 6 h among the carrageenan-treated groups, whereas expression remained elevated at 6 h in PBM-treated animals. In contrast to the reduction observed for IL-6, IP, and kinin receptors at specific time points, the maintenance of IL-10 expression suggests that PBM does not act exclusively by reducing inflammation-associated mediators but may also be associated with transcriptional responses related to regulatory mechanisms involved in the control of the inflammatory response.

The present study has limitations that should be considered when interpreting these findings. Saline-treated animals were evaluated only at 6 h; therefore, the Carr-1 and Carr-3 groups did not have corresponding time-matched saline controls, and changes at these earlier time points should primarily be interpreted as temporal differences among carrageenan-treated groups. In addition, a saline + PBM group was not included, precluding assessment of the effects of PBM alone under basal conditions. The experimental design also does not permit a formal assessment of a treatment × time interaction. Furthermore, the present findings are based on mRNA expression and were not complemented by protein-level or functional measurements. The use of total brain tissue does not allow identification of specific brain regions or cell populations involved in the observed transcriptional responses. In addition, 18 S rRNA was used as the sole reference gene, and its stability across tissues, experimental conditions, and time points was not formally evaluated. Finally, nociceptive behavior and direct measures of neuroinflammation or receptor activity were not assessed. The limited number of experimental time points should also be considered when interpreting the temporal profiles observed.

Taken together, the temporal changes observed in the paw and brain show that carrageenan-induced peripheral inflammation is accompanied by rapid changes in the expression of inflammatory and nociceptive mediators in the CNS. Local PBM modulated this response in both tissues, reducing IL-6, IP receptor, and kinin receptor mRNA expression at specific time points while maintaining elevated IL-10 expression. These findings extend previous observations from this experimental model by showing that the molecular effects associated with local PBM involve not only prostanoid and cytokine pathways but also components of the kinin–kallikrein system. Although the mechanisms linking local PBM to changes in brain gene expression remain to be established, the present results support a multi-target transcriptional response associated with PBM and show that its molecular effects are not restricted to the irradiated tissue.

## Data Availability

No datasets were generated or analysed during the current study.
